# Supplementary material for: Comparative Genomic Analysis of Virulent Vibrio (Listonella) anguillarum Serotypes Revealed Genetic Diversity and Genomic Signatures in the O-Antigen Biosynthesis Gene Cluster
Source: Microorganisms. 2023 Mar 20;11(3):792. doi: 10.3390/microorganisms11030792 (PMC10059132; doi:10.3390/microorganisms11030792)
Supplement: Supplementary file 1 [file microorganisms-11-00792-s001.zip › Supplementary file S1.pdf]

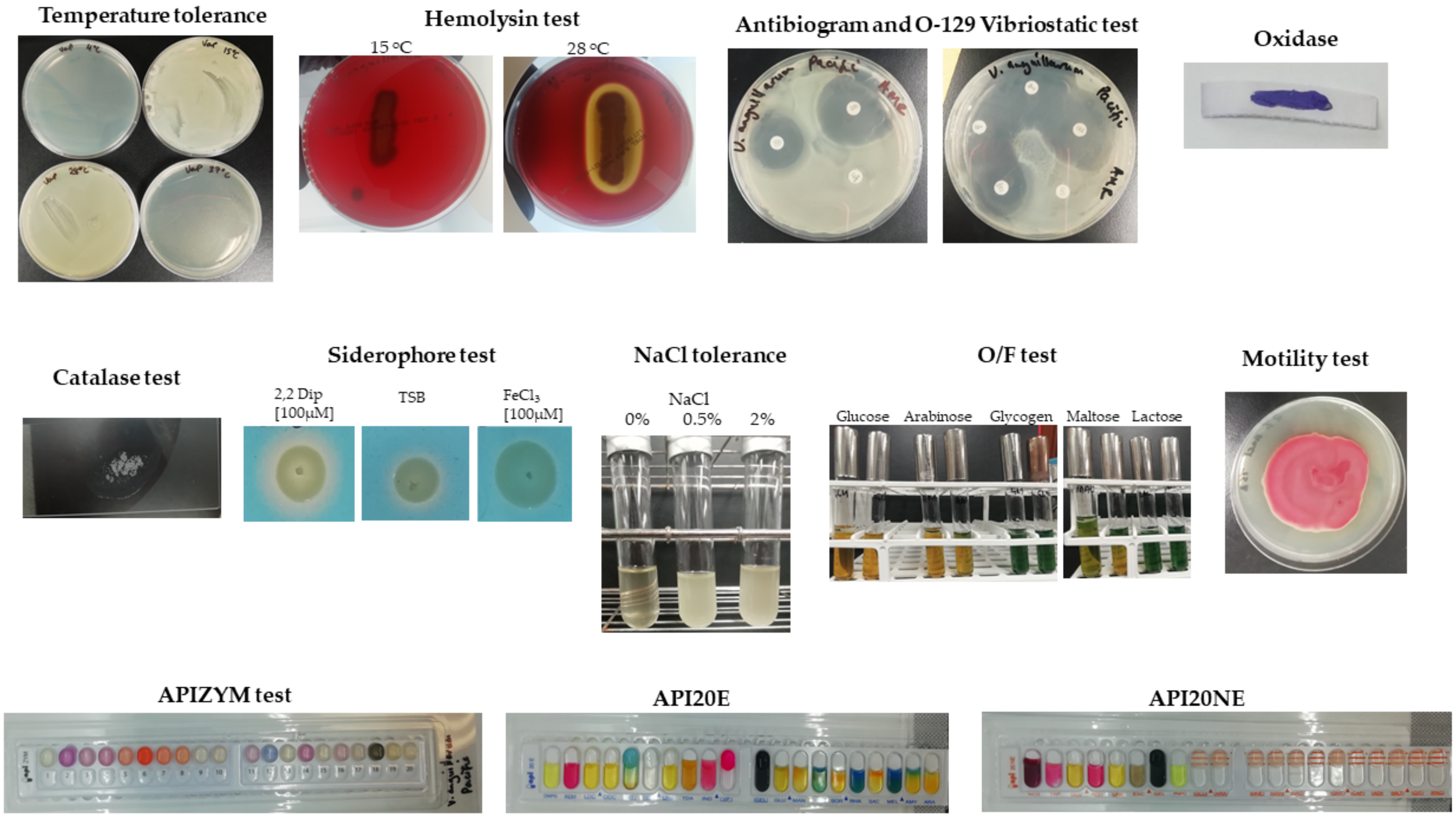

Figure S1. Phenotypic characterization tests conducted on *V. anguillarum* J382 isolated from winter Steelhead trout in British Columbia, Canada.

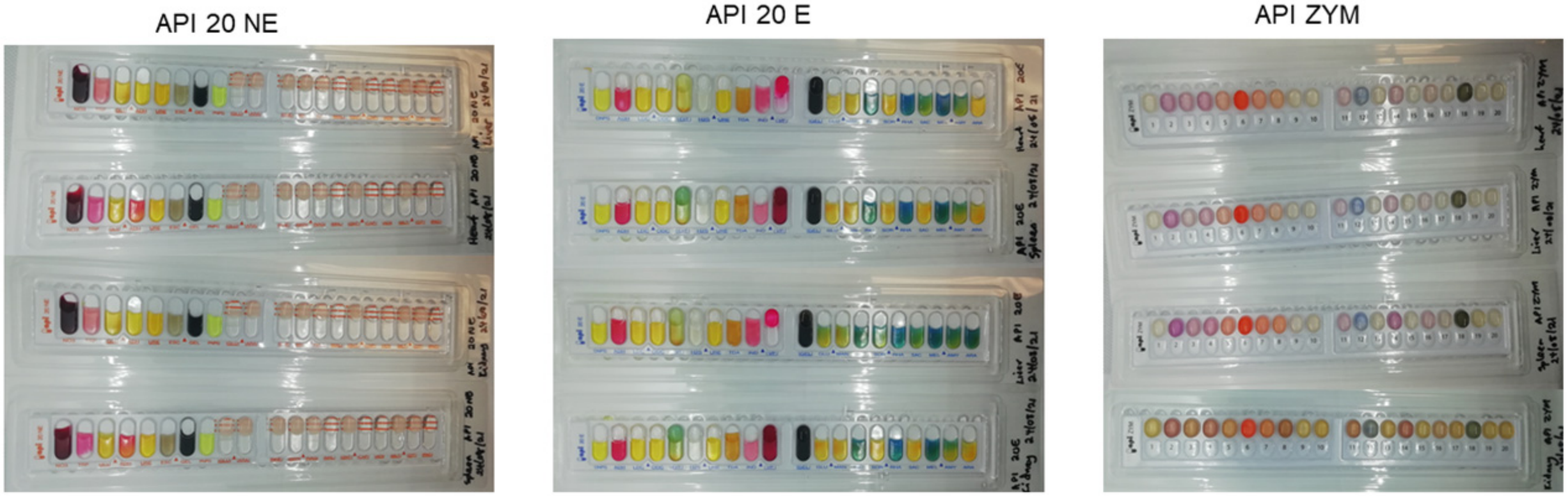

Figure S2. Characterization using API system of bacteria isolated from lumpfish organs (spleen, heart, liver, head kidney) infected with *V. anguillarum* J382 at 5dpi.

Table S1. Annotation summary of *V. anguillarum* J382 using Rapid annotation subsystem technology (RAST).

Characteristic	Chromosome 1	Chromosome 2	Plasmid pVa_O1-1	Plasmid pVa_O1-2
**Length**	3,133,133 bp	1,038,699 bp	65,573 bp	76,959 bp
**GC Content**	44.64	44.04	42.59	42.6
**CDS**	2,914	1,007	69	106
**tRNA**	95	4	0	0
**rRNA**	28	0	0	0
**Number of subsystems**	300	58	2	2

Table S2. Genes associated with pathogenesis and environmental adaption in *V. anguillarum* J382 predicted by RAST and Patric 2.0.

Subsystem Category	Gene Product	Presence/Absence of Gene			
	Feature	Chr1	Chr2	pVa_O1-1	pVa_O1-2
Iron acquisition and metabolism	PitA, PitC, PitD, YfeX, TonB, TolA, TolQ, Fur, TPR, TonBc, ExbD, Tmt1,<br>FhuA, ABC transporter, substrate-binding protein (cluster 8, B12/iron complex), PiuC, Iron transport protein, YbtT, Irp2,	X		X	X
Cell Wall and Capsule	RfbD, RfbF, TGDS, RfbC, RfbA, WbbL, GalE, TagH, TagG, CpsA, CpsB, CpsC, CpsD, LolA, LolC, LolE, AmpD, FtsI, GlmU, MurA, GlmM, AmpL, AmpG, WecG, IspD<br>GalE, ManB	X	 <br>X		
Motility and Chemotaxis	FlgA, FlgB, FlgC, FlgD, FlgE, FlgF, FlgG, FlgH, FlgI, FlgK, FlgL, FlgN, FlgO, FlgP, FlgT, FtsI, FliE, FliF, FliI, FliH, FliJ, FliN, FliO, FliP, FliR, FliS, FleN, FleQ, FleS, RpoD, RpoN, MotB, MotY, MotX, FlhB, FlaA, FlaC, FlaF,FlhA, FliQ, FliK, FliM, FlaD, FlhF, MotA, FliG, FleN, FlgH, CheV, FliN, FliR, RpoN, MotB, FlhA, FlgD, FliM, FlhB, FlhF<br>RNA polymerase sigma factor for flagellar operon	X			
Siderophore biosynthesis and transport	VibB, MenD, ExbB,<br>Siderophore biosynthesis protein, monooxygenase, non-ribosomal peptide synthetase modules, siderophore biosynthesis, MenF, EntA, EntB, EntD, ViuC, AroG	 <br>X		X	X
Secretion systems					
Type II secretion system	GspA, GspC, GspD, GspE, GspF, GspG, GspH, GspI, GspJ, GspK, GspL, GspM, GspN,	X			
Type IV pilus	PilA, PilB, PilC,PilD, PilE, PilM, PilN, PilO, PilP, PilQ, PilT, PilV,<br>N-methyltransferase (EC 2.1.1.-), AroB, MtgA, (EC 3.4.-.-)	X			
Toxins and superantigens	ToxR, ToxS, Ace, Zot<br><br>hypothetical toxin	X		 <br>X	 <br>X
Invasion and intracellular resistance	S5e, S23e, Tu, FusA, RpoB, NadA, NadB, NadC,	X	X		
Resistance to toxic compounds					
Copper homeostasis	CcmF, CcmH, CflA, copB, CopG, CutC, CutE, CorC	X			
Resistance to chromium compounds	ChrA	X			
Cobalt-zinc-cadmium resistance	CzcD, merR		X		

Table S2. Genes associated with pathogenesis and environmental adaption predicted by RAST and Patric 2.0. (continued)

Subsystem Category	Gene Product	Presence/Absence of Gene			
	Feature	Chr1	Chr2	pVa_O1-1	pVa_O1-2
Antibiotic resistance					
Resistance to fluoroquinolones	gyrA, gyrB	X			
Antibiotic activation enzyme	KatG	X			
Antibiotic target in susceptible species	Alr, dxr, EF-G, EF-Tu, folA, Dfr, folP, gyrA, gyrB, Iso-tRNA, kasA, MurA, rho, rpoB, rpoC, S10p, S12p	X			
Antibiotic target replacement protein	fabV	X	X		
Resistance to antimicrobial peptides	creA		X		
Gene conferring resistance via absence	gidB	X			
Protein altering cell wall charge conferring antibiotic resistance	GdpD, PgsA	X			
Regulator modulating expression of antibiotic resistance genes	H-NS, OxyR	X			
Multidrug Resistance Efflux Pumps	MATE_all, AcrB, Tet(35), TolC/OpmH	X			
Hemolysins	RbmC, hemolysin delta-VPH, Putative hemolysin, HylB	X			
	HlyA, hemolysin		X		

Table S3. Plasmids identical to *V. anguillarum* J382 plasmids pVA_O1_1 and pVA_O1_2 which were found after a blastn search in the NCBI database.

Plasmid		identical plasmid							
	length	ID	Accession	Max Score	Total Score	Query Cover	E value	Per. Ident	Acc. Len
pVA_O1_1	65573	*V. anguillarum* 775 plasmid pJM1	AY312585.1	53144	1.53E+05	100%	0	100.00%	65009
		V. anguillarum strain ATCC-68554 plasmid p65	CP023210.1	42826	1.53E+05	100%	0	100.00%	65009
		V. anguillarum strain Ba35-E2-2 plasmid pJM1	CP031522.1	32398	1.21E+05	100%	0	100.00%	64192
		V. anguillarum strain 425 plasmid pEIB1	CP020532.1	42815	1.56E+05	100%	0	99.99%	66521
		V. anguillarum strain 87-9-117 plasmid pJM1	CP016253.1	32398	1.23E+05	100%	0	100.00%	65072
		V. anguillarum plasmid p67vangNB10, strain NB10	LK021128.1	42789	1.58E+05	100%	0	99.97%	66798
		V. anguillarum strain BA35 plasmid pJM1 sequence	CP016261.1	32398	1.21E+05	100%	0	100.00%	64092
pVA_O1_2	76959	*V. anguillarum* strain ATCC-68554 plasmid p65	CP023210.1	57300	1.79E+05	100%	0	99.87%	65009
		V. anguillarum strain 425 plasmid pEIB1	CP020532.1	57289	1.81E+05	100%	0	99.87%	66521
		V. anguillarum 775 plasmid pJM1	AY312585.1	52730	1.79E+05	100%	0	99.68%	65009
		V. anguillarum strain Ba35-E2-2 plasmid pJM1	CP031522.1	33187	1.42E+05	100%	0	99.98%	64192
		V. anguillarum strain BA35 plasmid pJM1 sequence	CP016261.1	33187	1.42E+05	100%	0	99.98%	64092
		V. anguillarum strain 87-9-117 plasmid pJM1	CP016253.1	33178	1.44E+05	100%	0	99.98%	65072
		V. anguillarum plasmid p67vangNB10, strain NB10	LK021128.1	50848	1.84E+05	100%	0	99.83%	66798

Table S4. O-antigen biosynthesis gene cluster diversity among *V. anguillarum* serotypes O1, O2 and O3.

		87-9-116 (O1)		J382 (O1)		J360 (O2)		CNEVA NB 11008 (O3)	
EC #	Gene symbol	chr1	chr2	chr1	chr2	chr1	chr2	chr1	chr2
1.17.1.1	*asc*D/*ddh*D/*rfb*I	X		X					
4.2.1.45	*ddh*B/*rfb*G	X		X					
2.7.7.33	*ddh*A/*rfb*F	X		X					
2.7.7.9	*galU*	X		X		X		X	
1.1.1.22	*ugd*	X		X					
5.1.3.2	*gal*E	X	X	X	X		X	X	X
2.7.7.24	*rml*A	X		X				X	
5.1.3.6	*gla*/*gae*/*cap*1J	X		X					
4.2.1.46	*rml*B/*rfb*B	X		X				X	
5.1.3.13	*rml*C/*rfb*C	X		X					
1.1.1.133	*rml*D	X		X					
2.6.1.59	*wec*E/*rff*A	X		X					
5.3.1.8	*man*A	X		X		X		X	
5.4.2.8	*man*B		X		X		X		X
1.1.1.336	*mna*B/*wec*C/*vps*B	X		X		X		X	
5.1.3.14	*mna*A/*wec*B	X		X		X		X	
2.7.7.23/2.3.1.157	*glm*U	X		X		X		X	
4.2.1.135	*pgl*F/*wbp*M	X		X		X		X	
1.1.1.426	*wbp*V	X		X				X	
2.3.1.201	*wbp*I/*wlb*D	X		X		X			
2.6.1.102	*per*/*rfb*E	X		X					
1.1.1.136	*wbp*O					X		X	
2.6.1.98	*wbp*E/*wlb*C					X			
5.1.3.23	*wbp*I/*wlb*D/*wec*B					X			
1.1.1.335	*wlb*A/*wbpl*A					X			
5.1.3.7	*wbp*P/*tvi*C							X	
2.7.8.33	*wec*A	X		X					
1.17.1.1	*asc*C/*ddh*C/*rfb*H	X		X					

(a)

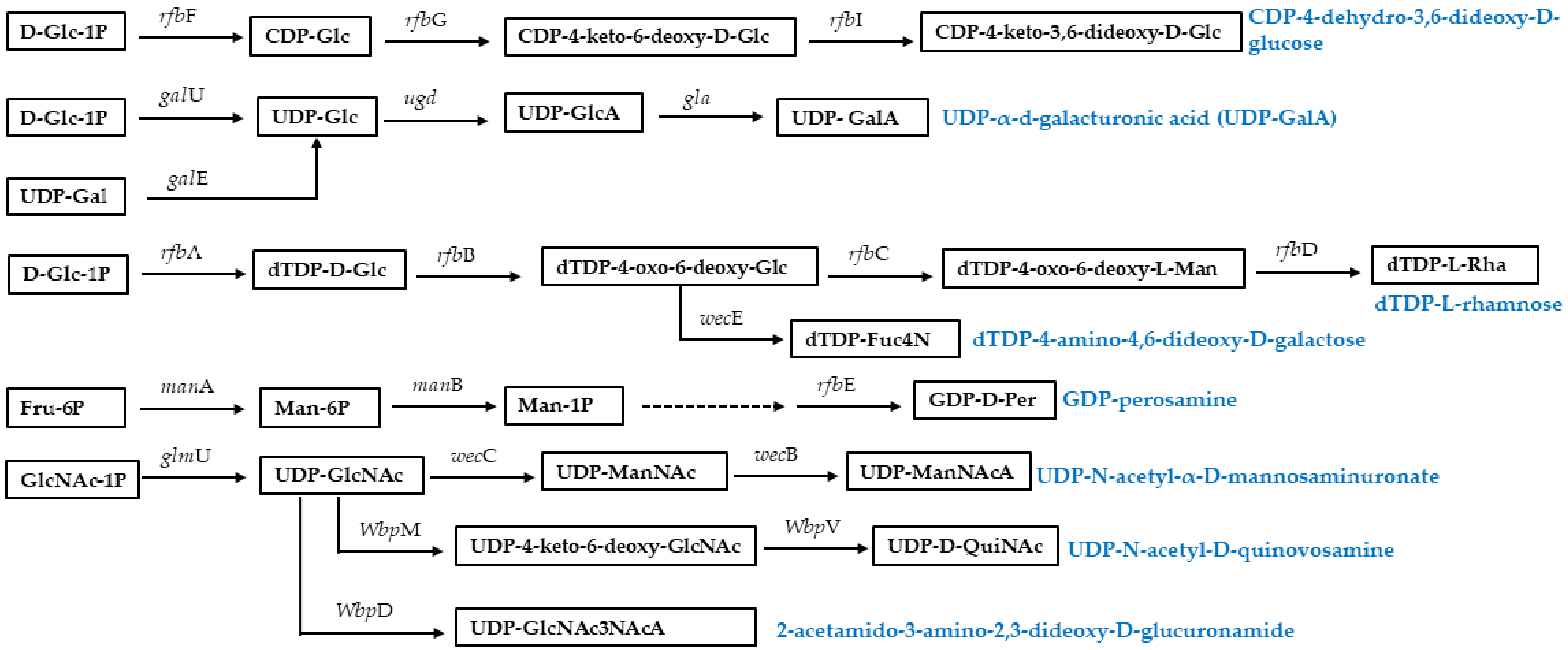

Figure S3(a). Putative O-antigen biosynthesis pathways associated with *V.anguillarum* serotype O1 and the corresponding oligosaccharides (blue color).

(b)

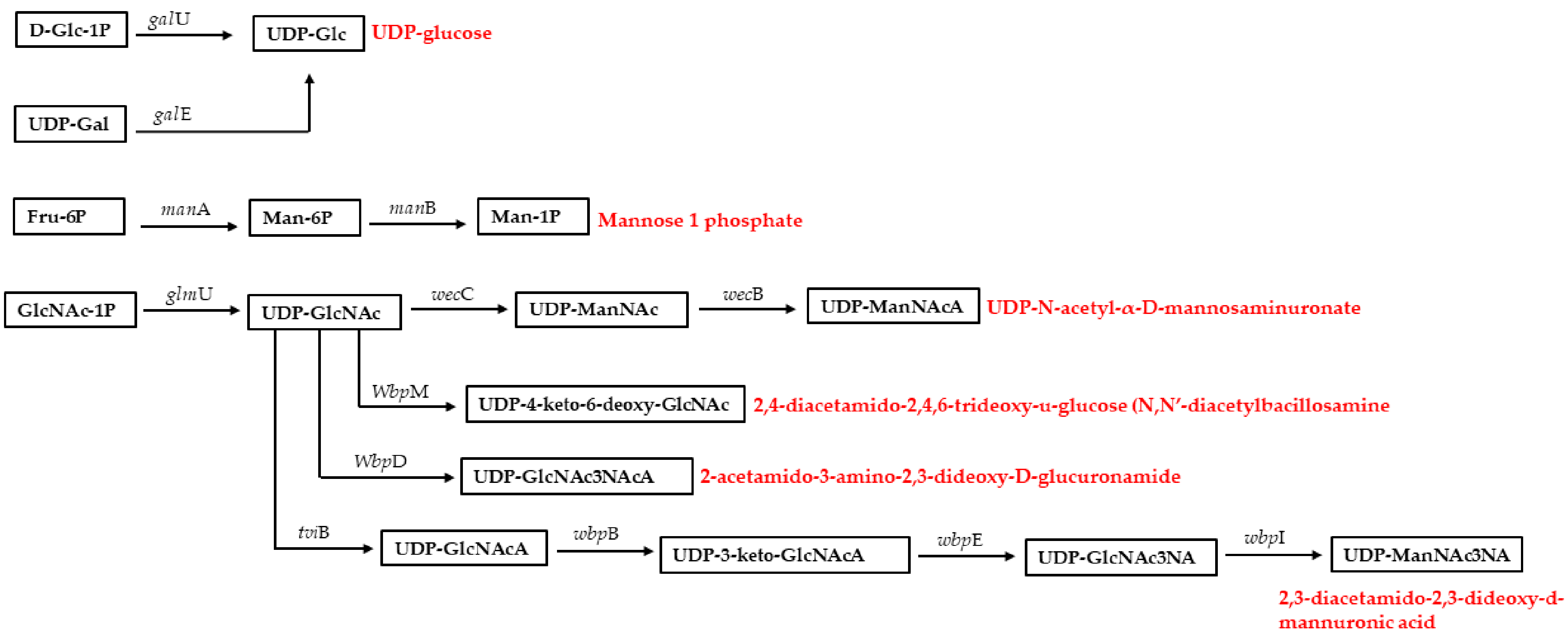

Figure S3(b). Putative O-antigen biosynthesis pathways associated with *V.anguillarum* serotype O2 and the corresponding oligosaccharides (red color).

(c)

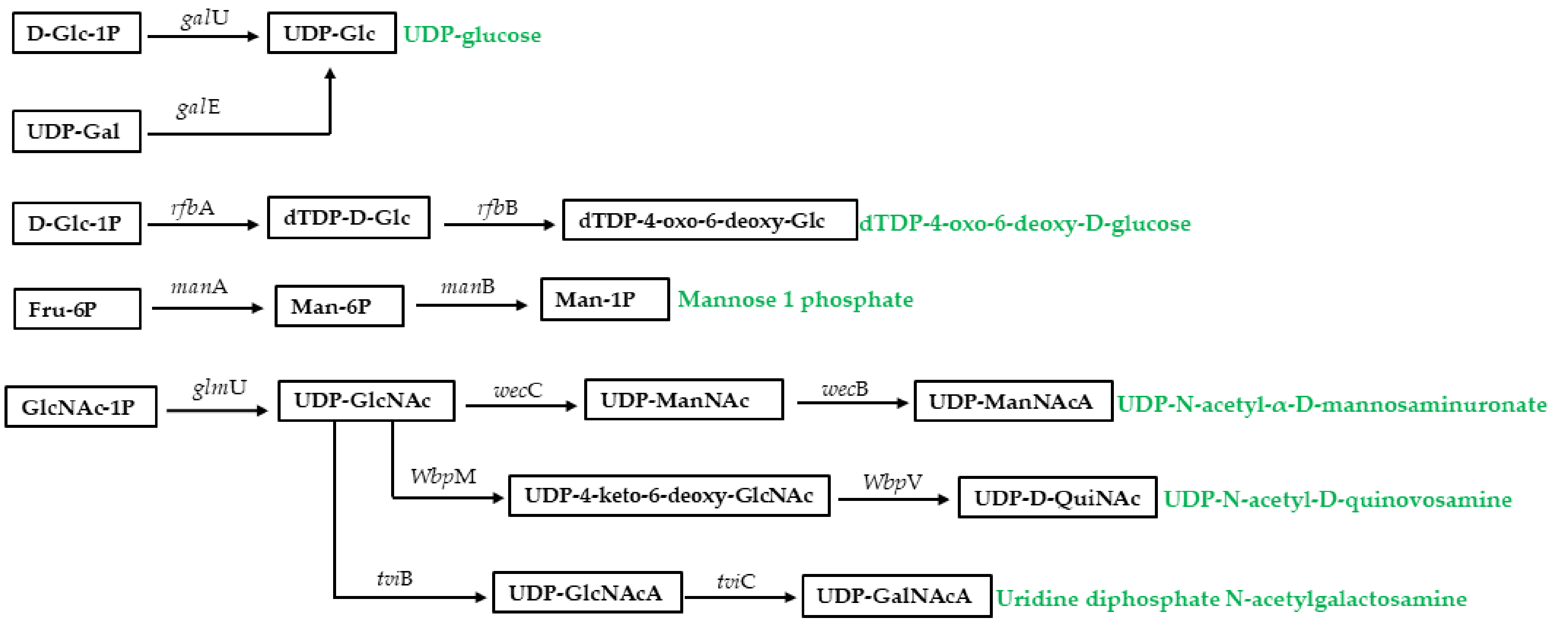

Figure S3(c). Putative O-antigen biosynthesis pathways associated with *V.anguillarum* serotype O3 and the corresponding oligosaccharides (green color).

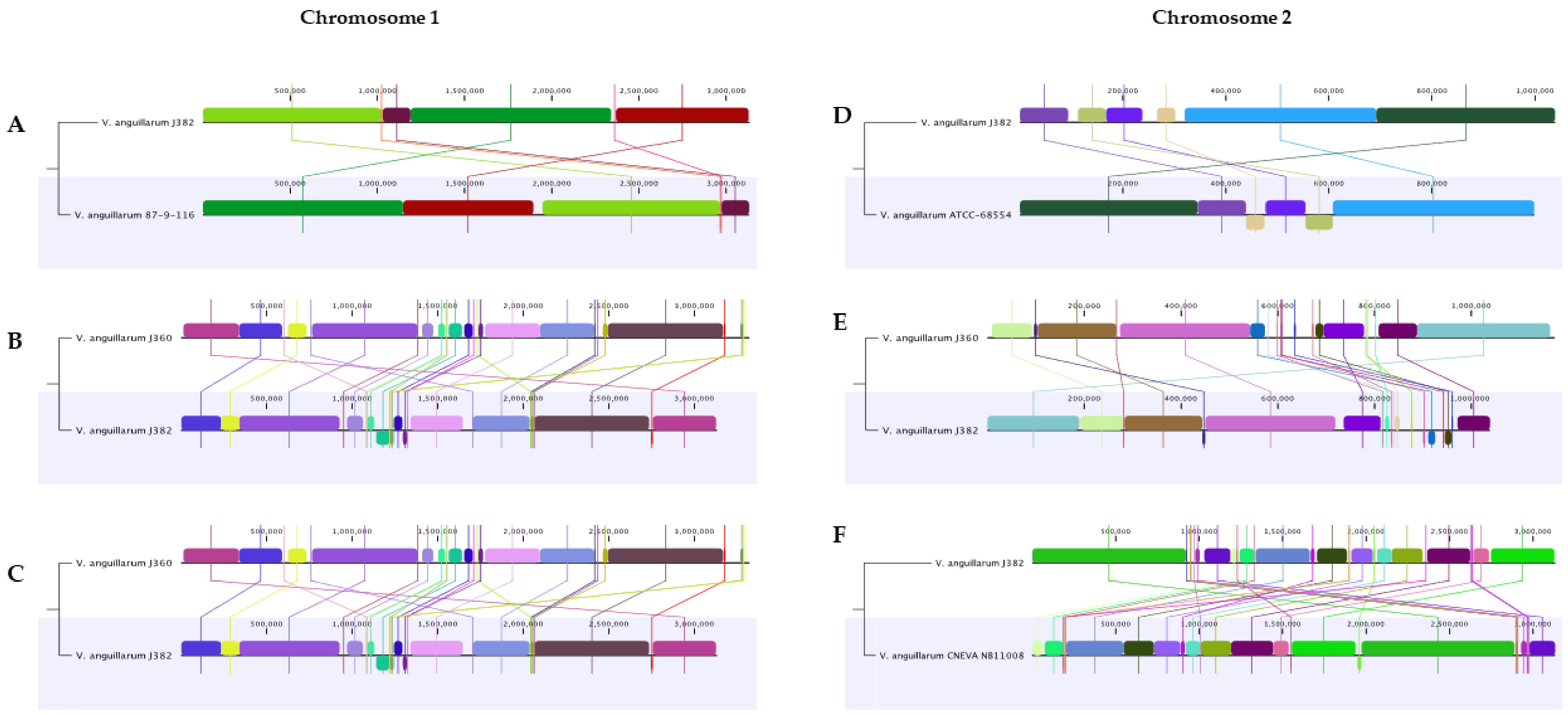

Figure S4. Synteny of locally colinear blocks between *V. anguillarum* J382 chromosome 1 and (a) serovar O1 V. *anguillarum* 87-9-116; (b) serovar O2 *V. anguillarum* J360; (c) serovar O3 *V. anguillarum* CNEVA NB 11008. Locally collinear blocks between *V. anguillarum* J382 chromosome 2 and (d) serovar O1 V. *anguillarum* 87-9-116; (e) serovar O2 *V. anguillarum* J360; (f) serovar O3 *V. anguillarum* CNEVA NB 11008.

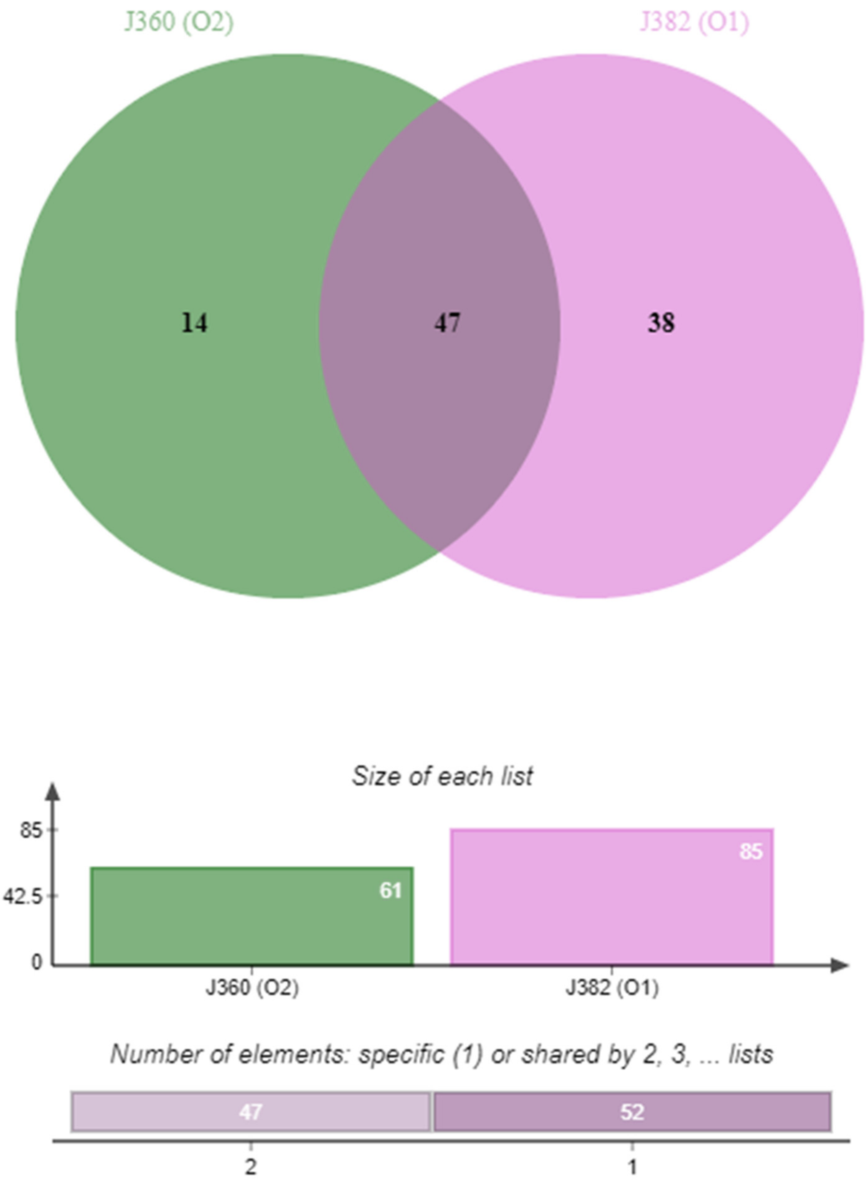

Figure S5. Comparison of ncRNAs in *V. anguillarum* serotypes O1 (J382) and O2 (J360). ncRNAs in *V. anguillarum* J382 were predicted using the software StructRNAfinder and BEDTools: intersect v2.3, while J360 ncRNAs were retrieved from the NCBI. ncRNA comparison was performed using jvenn software.
